# Meigs'‐Like Syndrome Secondary to Remnant Ovarian Tissue in a Cat

**DOI:** 10.1111/jvim.70168

**Published:** 2025-07-30

**Authors:** Clara Galvani, Silvia Bigi, Francesca Saponaro, Giulia Selmi, Matteo Ganapini, Maurizio Longo

**Affiliations:** ^1^ DIVAS—Department of Veterinary Medicine and Animal Science University of Milan Lodi Italy; ^2^ Castellarano Veterinary Clinic, Castellarano (RE) Castellarano Italy

**Keywords:** ascites, feline, ovarian remnant syndrome, ovary, pleural effusion

## Abstract

A 4‐year‐old spayed female domestic shorthair cat was evaluated for a three‐day history of dyspnea and lethargy. Abdominal ultrasonography and thoracic radiographs revealed the presence of abdominal and pleural effusions, along with both uterine horns and a rounded mass in the pelvic abdomen. Both effusions were compatible with modified transudates rich in protein and negative for infectious disease. The mass and uterus were surgically removed, and histology revealed a normal cycling ovary and uterine glandular hyperplasia. At the two‐week follow‐up after surgery, the effusion had completely disappeared, and the cat had fully recovered. In human medicine, Meigs' syndrome is characterized by the triad of pleural and abdominal effusions along with ovarian mass, most commonly fibromas. This is a report describing a Meigs' like syndrome in a young cat with ovarian remnant tissue.

**Abbreviations**
AMH: anti‐Müllerian hormone
aPTT: activated partial thromboplastin time
CA‐125: cancer antigen 125
FCoV: feline coronavirus
FCU: focused cardiac ultrasonography
FDPs: fibrin degradation products
FeLV: feline leukemia virus
FIP: feline infectious peritonitis
FIV: feline immunodeficiency virus
ORS: ovarian remnant syndrome
PT: prothrombin time
RI: reference interval
VEGF: vascular endothelial growth factor

## 1 Case Description

A 4‐year‐old, ovariectomized female, domestic shorthair cat was presented to the emergency service due to a three‐day history of dyspnea and lethargy. She had no significant health issues reported in her medical history, the only notable event being an ovariectomy at the age of 7 months. On presentation, the cat had a body condition score of 5/9, weighing 4.8 kg, and exhibited severe respiratory distress. On cardiothoracic auscultation, there were constant decreased heart and lung sounds bilaterally. The cat had a normal heart rate (180 beats/min), tachypnea (50 breaths/min), and normothermia (38.4°C). It was found to have pallid mucous membranes, with a normal capillary refill time.

Complete blood count (CBC) showed mild neutrophilia 8.829 × 10^9^/L (Reference Interval (RI): 2.5–7 × 10^9^/L). Biochemistry revealed minor alterations, including mild hypoproteinemia 5 g/dL (RI: 5.8–8 g/dL), mild hypoalbuminemia 2.3 g/dL (RI: 2.5–4 g/dL), mild hypoglobulinemia 2.7 g/dL (RI: 2.8–5.5 g/dL), and hyperglycemia 218 mg/dL (RI: 80–145 mg/dL). The blood gas analysis, urine analysis, and hemostatic profile (aPTT, PT, fibrinogen, FDPs, and antithrombin) were unremarkable.

**FIGURE 1:**
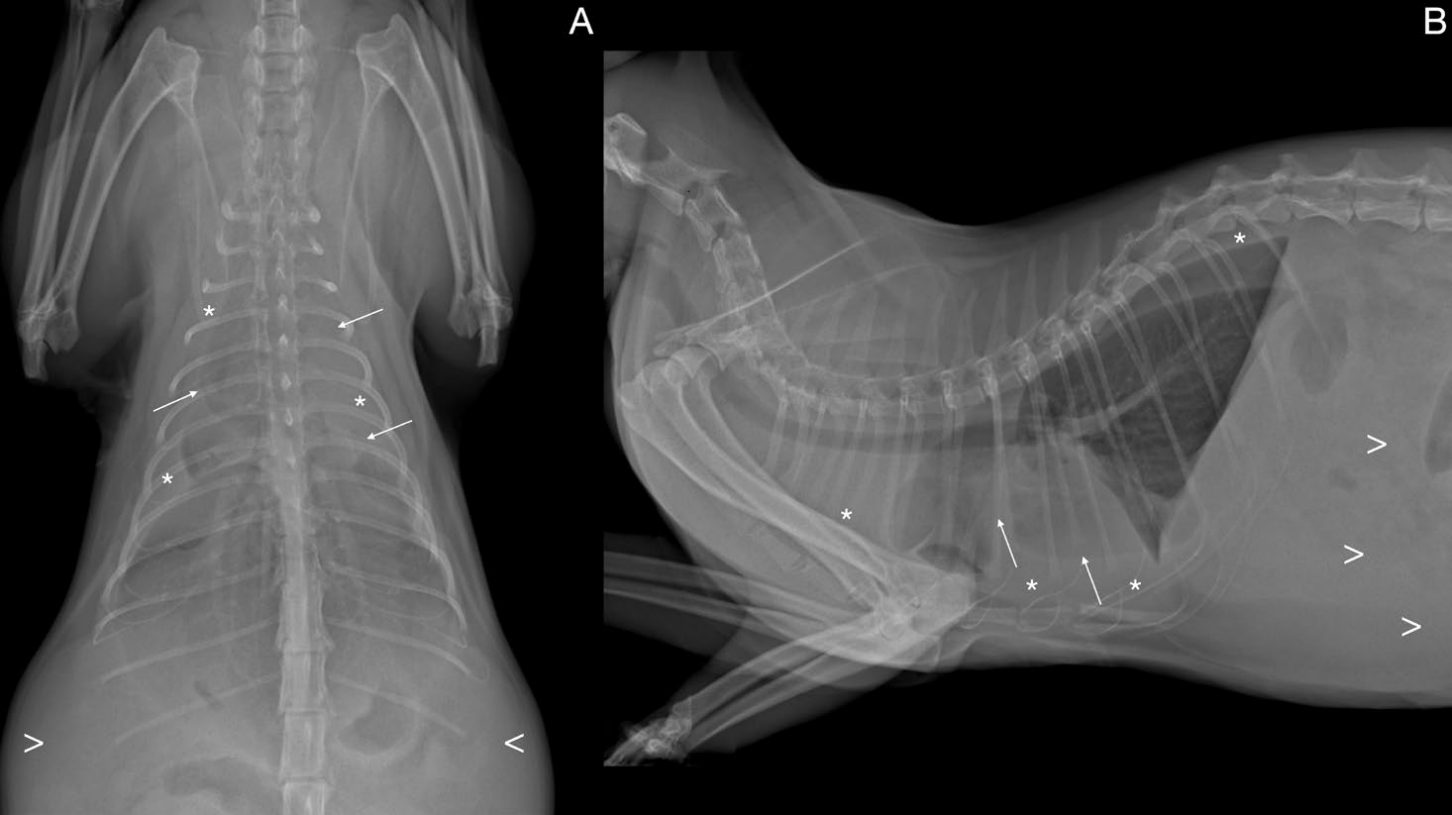
(A) Dorsoventral and (B) right lateral view of the thorax with pleural effusion (asterisk) and secondary lung atelectasis (arrow) in the cat presented with severe respiratory distress. Visible reduced detail of the cranial abdomen (arrowhead) due to the presence of abdominal effusion.

**FIGURE 2:**
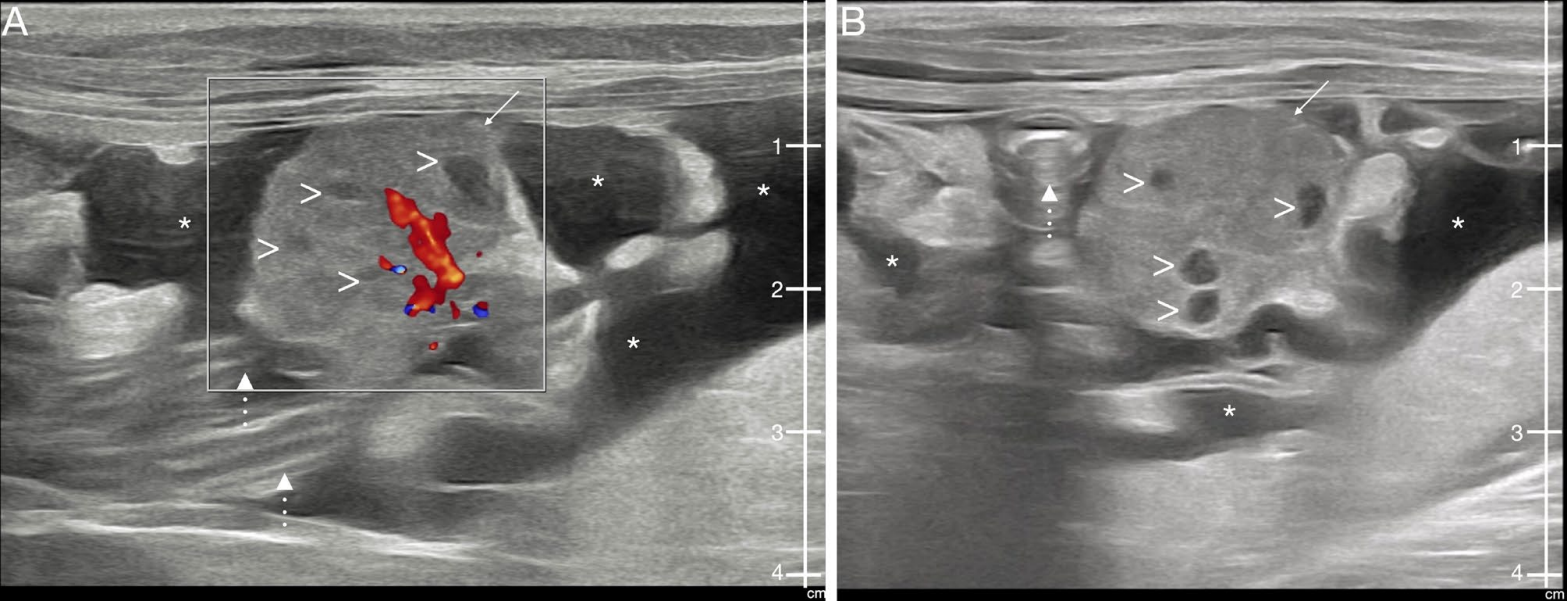
Ultrasound B‐mode long axis images obtained with linear probe (MHz 12) with mixed Color Doppler flow (A), and without (B), in the female cat presented with respiratory distress, to evaluate the cause of abdominal effusion. The image shows the abdominal mass (remnant ovarian tissue; arrow) featuring multiple hypoechoic areas internally (arrowhead), abdominal effusion (asterisk), and intestinal loops (dotted line).

Thoracic radiographs were obtained as the initial imaging modality. The orthogonal projections depicted a moderate quantity of pleural effusion with secondary pulmonary atelectasis and reduced abdominal detail in the cranial abdomen, suggestive of abdominal effusion (Figure 1). For further investigations, abdominal ultrasound (Samsung V8) with a convex (3–10 MHz) and linear probes (2–14 MHz) was performed under sedation. Butorphanol (0.2 mg/kg IM, Torphadine, Dechra S.r.l., Turin, Italy) and Dexmedetomidine (8 μg/kg IM, Dexdomitor, Vétoquinol Italia S.r.l., Bertinoro, Italy) were used as premedication and Propofol as dose‐effect IV (Propomitor, Ecuphar Italia S.r.l., Milan, Italy). Ultrasonographic examination revealed a moderate amount of anechoic abdominal effusion. Both uterine horns were present, and a heterogeneous rounded mass with a maximum diameter of 1.8 cm, featuring multiple hypoechoic areas internally, was observed cranial to the urinary bladder (Figure 2). This mass showed abundant vascularization on Color Doppler examination and was not in clear communication with the uterine horns. The rest of the abdominal organs showed no abnormalities.

Although under sedation, the abdominal and thoracic effusions were sampled and drained using a sterile 21‐gauge wedge infusion set. Approximately 180 mL of pleural effusion and 20 mL of abdominal effusion were drained. Subsequently, the abdominal mass was sampled using a sterile 22‐gauge needle. The analysis of the effusions revealed that both were consistent with modified transudates rich in proteins. Due to the critical condition of the cat, a focused cardiac ultrasonography (FCU) evaluation was performed to rule out a cardiac origin. The exam revealed normal cardiac morphology, appropriate chamber sizes, and no evidence of pericardial effusion. PCR for *Coronavirus* (FCoV/FIP) on pleural fluid and mass aspirate was negative, as were the FIV/FeLV ELISA test (SNAP Combo Plus, IDEXX Laboratories, Westbrook, ME, USA) and culture of pleural effusion (on eSwab for aerobic, anaerobic, and fastidious bacteria, COPAN, Brescia, Italy). The cytology of the mass was suggestive of a hyperplastic/neoplastic lesion of epithelial origin. The cat was temporarily discharged with Bromelain (5 mg/kg p.o. q24h, Fortilase, Rottapharm, Monza, Italy). At the 5‐day follow‐up, the cat's clinical condition was improved, and the respiratory rate was within normal limits. On radiographic and ultrasonographic examinations, pleural effusion was minimal, whereas ascites mildly increased compared to the day of the discharge. The mass remained unchanged. Based on the suspicion of a hyperplastic/neoplastic lesion on cytology, the mass was surgically removed the following day. A midline surgical approach was performed, revealing a moderate quantity of abdominal effusion. A well‐defined, rounded mass was identified attached to the uterus but located more centrally compared to the normal anatomical position of the ovaries. The mass was associated with peritoneal adhesions and fibrotic tissue, incorporated with a nonabsorbable braided surgical thread. Careful dissection was performed to completely excise the mass along with both uterine horns.

The histology of the mass consisted entirely of ovarian parenchyma, featuring numerous corpus lutea and follicles at various stages of maturation with no evidence of neoplastic cells. The endometrium of the uterus showed diffusely and moderately hyperplastic endometrial glands with mild edema, whereas the myometrium was slightly expanded due to mild edema.

The histological findings were consistent with a cycling ovary and diffuse, moderate glandular hyperplasia of the uterus.

At two‐week follow‐up after surgery, the effusions had completely disappeared and the cat was fully recovered.

## 2 Discussion

Ovarian remnant syndrome (ORS) is a frequent condition in veterinary medicine, especially in cats, but to the authors' knowledge this has not been described in association with bicavitary effusion. ORS is a result of failure to remove the entire ovary during spaying, and it can be characterized by clinical signs related to the functional ovarian cycle. It can be symptomatic, mostly with estrus and proestrus behaviors, or asymptomatic [1]. The visualization of ovarian tissue on diagnostic imaging, notably on ultrasonographic examination, sometimes is challenging because the residual tissue might present a limited volume, especially if the animal is in anestrus. If the tissue is visible, it appears as normal ovarian tissue of variable size with or without follicles or corpora lutea. Its location is not always at ovarian pedicles; indeed, it can be displaced to other locations inside the abdominal cavity during surgery, but it has the potential to revascularize and return to its endocrine function [2]. Anti‐Müllerian hormone (AMH), secreted by granulosa cells or Sertoli cells, is the current gold standard for the diagnosis of ORS [3]. In our case, the cat underwent ovariectomy at the age of 7 months and did not exhibit any heat‐like behavior in her history.

Meigs' syndrome is a condition depicted in human medicine since 1954 and characterized by the triad of benign ovarian mass, mostly fibromas or fibroma‐like tumor such as thecoma, granuloma cell tumors, and Brenner tumors along with ascites and pleural effusions [4, 5]. The resolution of clinical signs after gonadal removal is the key feature of Meigs' syndrome.

In human medicine about 1% of ovarian tumors might be associated with Meigs' syndrome [6]. In literature this condition was further classified in different types, mainly as Demons‐Meigs', pseudo‐Meigs', and pseudo‐pseudo Meigs' syndrome, depending respectively on the type and location of the tumors involved. Demons‐Meigs' syndrome includes benign genital tumors other than fibromas or fibromas‐like [5, 7]; pseudo‐Meigs' includes benign and malignant tumors, including metastasis, in the caudal abdomen outside of the genital tract [5, 8, 9]; pseudo‐pseudo Meigs' syndrome, also known as Tjalma syndrome, is described in a patient with systemic lupus erythematosus, ascites, pleural effusion, and raised carbohydrate antigen 125 (also known as cancer antigen 125, CA‐125) [10].

To avoid confusion, in the present case, as the ovarian tissue was normal, the authors referred to this condition as Meigs'‐like syndrome, considering the absence of neoplastic tissue on histopathology.

The exact pathophysiology of Meigs' syndrome is still unknown. In the present case, the ovary was increased in volume without the presence of neoplastic tissue. Therefore, the most likely hypothesis is lymphatic and venous obstruction associated with a voluminous ovary [9]. Indeed, the size of the ovary was quite large, measuring 1.8 cm in diameter, compared to the median length on ultrasound reported in the literature of 0.71–1.39 cm [11]. This is particularly notable, as only residual ovarian tissue was expected to remain, but it is uncertain whether part of the ovary was removed or if the entire organ was left intact during the previous neutering. Pleural effusion is highly suspected to have formed secondary to ascites, thorough transdiaphragmatic transport, or via lymphatic drainage, as its quantity was minimal 5 days after thoracic and abdominal drainage [6]. At the 7‐month follow‐up, the cat remains healthy, and no effusions have recurred.

A minor limitation of this case report is the lack of a complete echocardiography examination. However, despite this, the clinical signs resolved without the need for cardiac treatment, and the FCU evaluation suggested that heart function was within normal limits.

In conclusion, although the ovary was not consistent with a fibroma, our case shares similarities with the well‐known human Meigs' syndrome. This condition is benign, and prompt intervention is associated with a good prognosis. Furthermore, we can speculate that this rare condition might represent a complication of ORS in veterinary medicine.

## Disclosure

Authors declare no off‐label use of antimicrobials.

## Ethics Statement

Authors declare no institutional animal care and use committee or other approval was needed. Authors declare human ethics approval was not needed.

## Conflicts of Interest

The authors declare no conflicts of interest.
